# Context-Dependent Fitness Trade-Offs in *Penicillium expansum* Isolates Resistant to Multiple Postharvest Fungicides

**DOI:** 10.3390/microorganisms13081846

**Published:** 2025-08-07

**Authors:** Jonathan T. Puglisi, Achour Amiri

**Affiliations:** Department of Plant Pathology, Tree Fruit Research and Extension Center, Washington State University, 1100 N. Western Ave., Wenatchee, WA 98801, USA; jonathan.puglisi@wsu.edu

**Keywords:** pome fruit, blue mold, fitness, gene expression, fludioxonil, pyrimethanil, thiabendazole, cumulative fitness

## Abstract

Blue mold of pome fruit, caused by *Penicillium expansum*, is controlled through postharvest applications of thiabendazole (TBZ), pyrimethanil (PYR), and fludioxonil (FDL). However, multi-fungicide-resistant isolates have emerged in the U.S. Pacific Northwest and their impact on decay control in long-term storage is unknown. This study evaluated the fitness of *P. expansum* isolates sensitive to all three postharvest fungicides (wild-types) and those resistant to TBZ (single-resistant), TBZ and PYR, or PYR and FDL (dual-resistant), and triple-resistant to the three fungicides. On nutrient-poor media, resistant isolates showed reduced conidial germination, whereas no significant differences were observed in germination, mycelial growth, or sporulation between phenotypes on nutrient-rich media at 1.5 and 20 °C. Regardless of their sensitivity phenotype, FDL-resistant isolates showed increased sensitivity to osmotic and oxidative stresses. Pathogenicity and virulence were not affected by the sensitivity phenotype on apples after six months of storage at 1.5 °C. Analysis of cumulative fitness changes indicated fitness loss under low-temperature in vitro and increased fitness under fungicide selection pressure on fruit in most resistant phenotypes. Gene expression analysis showed differential regulation of fitness-related genes, with most being up-regulated by TBZ. Overall, the results suggest that resistance in *P. expansum* may carry context-dependent fitness penalties, especially under high-stress conditions.

## 1. Introduction

Blue mold, caused by *Penicillium expansum* (Link) and other *Penicillium* spp., represents the most significant postharvest disease affecting pome fruit globally [1,2,3,4] and is estimated to account for approximately 50% and 30% of decayed apples and pears, respectively, in the U.S. Pacific Northwest (PNW) [5,6]. To effectively manage blue mold, rigorous sanitation practices in packinghouse facilities are essential to reduce the initial level of *Penicillium* spp. inoculum, which may significantly influence the incidence of blue mold during storage [7]. Alongside sanitation measures, chemical control remains the main approach for managing blue mold and other postharvest diseases. Thiabendazole (TBZ) has been used for over five decades, whereas pyrimethanil (PYR) and fludioxonil (FDL) have been used postharvest in packinghouses for over two decades in the PNW and other growing regions. *Penicillium expansum* is classified as a medium-risk fungus for fungicide resistance development [8].

The repeated application of fungicides with the same mode of action has led to the emergence of resistant fungal populations. Consequently, the resistance of *P. expansum* to TBZ (Fungicide Resistance Action Committee, FRAC 1), PYR (FRAC 9), and FDL (FRAC 12) has emerged in the PNW [9,10,11], as well as in other pome fruit growing regions [12,13,14]. The resistance of *P. expansum* to FRAC 1 fungicides was first documented in the late 1970s [15]. Specific mutations at codons 167, 198, and 200 in the target β-tubulin gene of *P. expansum* have been reported to confer resistance to TBZ [12,13,16,17]. Currently, the FRAC1 fungicides thiophanate-methyl and TBZ are still sprayed pre- and postharvest, respectively, in the PNW. In the early 2000s, the anilinopyrimidine PYR and the phenylpyrrole FDL were registered to control postharvest diseases in packinghouses. Although the precise mechanism of action of PYR against *P. expansum* is not adequately elucidated, it is posited to inhibit enzyme secretion in other fungal species [18,19], disrupt methionine biosynthesis [20], and interfere with mitochondrial functions [21]. Instances of PYR resistance in *P. expansum* from the PNW and Mid-Atlantic regions were first reported a decade after its registration [11,12] and have increased in subsequent years [9,22]. Resistance of *P. expansum* to cyprodinil, another FRAC 9 fungicide, was also reported in Greece [23,24]. By comparison, FDL is believed to induce the synthesis of polyols, specifically glycerol and mannitol, resulting in abnormal hyphal morphology and cell lysis [25,26]. Resistance to FDL has been reported in *P. expansum* isolates from the U.S. PNW and Mid-Atlantic regions [9,27] as well as in Greece [23,24]. Resistance to FDL in *Penicillium* spp. has been associated with deletions or substitutions in the *OS-2* and *NikA* genes, a part of the group III histidine kinase of the high-osmolarity glycerol pathway [9,18,26,28,29]. The increasing prevalence of *P. expansum* populations resistant to TBZ, PYR, and FDL in packinghouses highlights the importance of comprehending how the evolution of fungicide resistance influences the relative fitness of these populations and its impact on decay management in long-term storage.

The fitness of filamentous fungi is attributed to their capacity to survive and reproduce in niches with a relatively low density of unoccupied space, while concurrently maximizing spore production [30]. Fluctuating impacts have been documented on the phenotypic fitness of *P. expansum* in relation to the development of fungicide resistance. Thus, isolates with single resistance to TBZ increased sporulation in vitro [9] and increased virulence on detached pears [12]. Conversely, PYR-resistant isolates of *P. expansum* demonstrated no significant differences in virulence on detached apples compared to PYR-sensitive isolates [13]. Laboratory mutants of *P. expansum* with single resistance to FDL showed decreased growth and increased sensitivity to osmotic stress in vitro, as well as decreased virulence and sporulation on apples [31]. Moreover, *P. expansum* field isolates with dual- or multiple-resistance to several preharvest fungicides used in Greek apple orchards displayed significantly decreased mycelial growth and pathogenicity on detached apples in short-term storage at room temperature, but their sporulation was greater than that of the wild-type isolates [23,24].

Fitness in filamentous fungi is often assessed at the phenotypic level by measuring traits such as spore germination, mycelial growth, sporulation, pathogenicity, virulence, and mycotoxin production. However, fitness metrics are regulated by global transcription factors that coordinate key physiological processes during infection. For example, *LaeA* regulates secondary metabolism such as patulin production and forms a complex with *VeA* to control virulence and development [32,33]. The velvet protein family, comprising *VeA*, *VelB*, *VosA*, and *VelC*, governs conidiation, secondary metabolism, and stress responses. *VeA* mutants exhibited defects in spore formation and failed to produce patulin and citrinin [32]. Additionally, *Blistering1* plays a pivotal role in virulence, mediating vesicle-based secretion of cell wall-degrading enzymes. Mutants of *P. expansum* deficient of *Blistering1* exhibited reduced virulence on apples [34]. Meanwhile, the transcription factor *brlA* is essential for conidiophore development and required for asexual reproduction. *P. expansum* mutants deficient in *brlA* lacked spore production but showed increased virulence [35]. In contrast, *Pdac1*, an adenylyl cyclase, regulates hyphal growth, virulence, and spore germination. *Pdac1*-deficient mutants produced heat-sensitive conidia with delayed germination [36]. Stress response pathways are also critical for *Penicillium* spp. pathogenicity. *OS*-2, homologous to *Hog1*, is a mitogen-activated protein kinase involved in osmotic stress tolerance. In *P. digitatum*, *OS-2* mutants exhibited reduced mycelial growth and glycerol production under salt and sorbitol stress and showed altered sensitivity to fludioxonil [25,26]. However, in *P. expansum*, the role of *OS-2* in osmotic stress response is less clear [37]. Additionally, the NADPH oxidase gene *PeRacA* regulates reactive oxygen species (ROS) production, growth, and pathogenicity. *PeRacA*-deficient mutants showed reduced growth under oxidative stress, highlighting the gene’s role in stress tolerance and infection success [38]. Collectively, these regulatory networks demonstrate the complex interplay between fungicide resistance, stress adaptation, and virulence in *Penicillium* spp. Assessing how fungicide resistance evolution affects the expression of key genes in *P. expansum* populations with different fungicide sensitivity phenotypes would provide valuable insights into phenotypic fitness and support the development of sustainable blue mold management strategies in pome fruit. Therefore, this study aimed to evaluate (i) fitness capabilities in vitro, (ii) virulence, sporulation and resistance stability on apples, and (iii) relative expression of several fitness-related genes and transcription factors in *P. expansum* isolates with single, dual, and triple resistance to TBZ, PYR, and FDL associated with blue mold outbreaks in packinghouses in the U.S. PNW.

## 2. Materials and Methods

### 2.1. Selection of Penicillium expansum Isolates with Different Fungicide Sensitivity Phenotypes

Fifteen *P. expansum* isolates with different fungicide sensitivity phenotypes were used in this study (Table 1). The isolates were obtained from decayed apples and pears collected from multiple warehouses in Washington State and Oregon between 2016 and 2018. The isolates were single-spored, stored as spore suspensions in 20% glycerol at −80 °C, and reactivated on potato dextrose agar (PDA) plates for 7 days at 20 °C prior to each experiment. The single-spored *Penicillium* isolates were characterized to the species level using a multi-locus sequence analysis as described previously [9,10,39].

The sensitivities of the single-spored isolates to TBZ (Mertect 340F, 42.3% a.i., Syngenta Crop Protection, Research Triangle, Greensboro, NC, USA) and FDL (Scholar, 20.46% a.i., Syngenta Crop Protection, Greensboro, NC, USA) were evaluated on PDA amended with 10 and 0.5 µg mL^−1^, respectively, and on sucrose agar (SA) amended with 0.5 µg mL^−1^ of PYR (Shield-Brite Penbotec 400 SC, 37.14% a.i., Pace International, Wapato, WA, USA). These doses were previously developed to discriminate resistant from sensitive isolates [40,41]. Non-amended and fungicide-amended plates were inoculated with three 10 µL droplets of spore suspensions of each isolate at 10^5^ conidia mL^−1^ in 0.05% Tween-20 and incubated for 24 h at 20 °C. Concentrations of spore suspensions were determined using a hemacytometer (Hausser Scientific, Horsham, PA, USA). Two replicate plates (six values) were used for each isolate and fungicide combination. The germination and length of the germ tubes were determined microscopically, and data were used to calculate percentage germination. Conidia exhibiting germ tubes that were twice as long as the conidium diameter indicated resistance to the specific fungicide. The trial was conducted once at the onset of each parameter investigation.

Phenotyping yielded three isolates sensitive to all three postharvest fungicides, three with single resistance to TBZ (TBZ^R^), three isolates with dual resistance to TBZ and PYR (TBZ^R^PYR^R^), three with dual resistance to PYR and FDL (PYR^R^FDL^R^), and three isolates with triple resistance (TBZ^R^PYR^R^FDL^R^) to all three fungicides (Table 1). Additional screenings to identify *P. expansum* isolates with single resistance to PYR (PYR^R^) and FDL (FDL^R^) or dual TBZ-FDL resistance (TBZ^R^FDL^R^) were unsuccessful.

### 2.2. Evaluation of Fitness In Vitro

Six fitness parameters, i.e., conidial germination, mycelial growth, sporulation, sensitivity to osmotic stress (OSS), and reactive oxygen species (ROS), as well as the stability of the sensitivity phenotypes, were evaluated for the 15 *P. expansum* isolates at two distinct temperatures to assess fitness capabilities of the isolates under various conditions. Conidial germination was assessed on PDA, 2% water agar (WA), and intermediate medium (IM; 2 g MgSO_4_·7H_2_O, 4 g glucose, 4 g peptone, 0.75 g KH_2_PO_4_, 2 g citric acid, 12 g agar, and 0.2 mL Tween 20 in 1 L of distilled water) [38]. Three 10 µL droplets of each isolate, at 10^5^ spores mL^−1^, were deposited equidistantly in a triangular arrangement on each plate and the plates were sealed with parafilm and incubated in the dark at 1.5 or 20 °C. Spore germination was assessed after 24 h on PDA and IM and after 48 h on WA at 20 °C. Germination at 1.5 °C was assessed after 24, 48, 72, and 96 h on PDA and IM and up to 10 days on WA. A total of 100 spores were examined by placing the plates upside down under a microscope and data were used to calculate the percentage of germination.

The sealed plates used for germination assessment were used to assess mycelial growth except on WA. Colony diameter was measured after 5 and 10 days of incubation at 20 °C and at 20-day intervals for up to 120 days at 1.5 °C in the dark. The sporulation on PDA and IM at 20 and 1.5 °C was assessed after 15 and 120 days, respectively, on the same plates used for mycelial growth assay. Ten milliliters of sterile distilled water with 0.05% Tween 20 were added to each plate and scraped using a sterile inoculation loop. Spore concentration was determined using a hemacytometer (Hausser Scientific, Horsham, PA). The sensitivities to OSS and ROS were assessed at 20 and 1.5 °C on PDA amended with 12% NaCl and 30 mM of methyl viologen (Mv, Acros Organics, Fair Lawn, NJ, USA), respectively, following the methodology used for the mycelial growth assay. For the above fitness parameters, three replicate plates were used for each isolate, medium, and temperature treatment combination across two trials.

The stability of fungicide sensitivity phenotypes of the 15 *P. expansum* isolates in the absence of fungicide selection pressure was assessed on PDA at 1.5 and 20 °C. In the 20 °C trial, conidia were prepared as described previously [42] and were transferred weekly to fresh PDA for 10 weeks. Due to the absence of sporulation at 1.5 °C, stability was evaluated through monthly transfers of 5 mm mycelial plugs taken from the edge of 30-day-old colonies grown on PDA. The plugs were placed upside down on fresh PDA plates and transferred for 10 consecutive months. Isolates subjected to 10 weekly transfers at 20 °C or 10 monthly transfers at 1.5 °C were designated as G_w_10 and G_m_10, respectively, while G0 referred to the original isolates before any transfer. To assess potential shifts in fungicide sensitivity between G0 and G10, the effective concentrations required to inhibit 50% germination and growth (EC_50_) were determined. Sensitivity to TBZ and FDL was assessed on PDA amended with TBZ at 0.0, 1, 5, 10, 50, 100, and 500 µg mL^−1^ and FDL at 0.0, 0.05, 0.5, 1, 5, 10, and 50 µg mL^−1^. Pyrimethanil sensitivity was assessed through germination inhibition on SA and mycelial growth inhibition on L-Asparagine agar (1 g K_2_HPO_4_; 1 g MgSO_4_; 0.5 g FeSO_4_·7H_2_O; 2 g L-Asparagine; 2 g glucose; 22 g agar) amended with PYR at 0.0, 0.05, 0.5, 1, 5, 10.0, and 50 µg mL^−1^. Three replicate plates were used for each isolate and the test was conducted once. Plate inoculation was conducted following previously described methods [9,43], spore germination and mycelial growth were evaluated after 24 h and 5 days, respectively, and data were used to calculate EC_50_ values as described previously [9,10].

### 2.3. Evaluation of Fitness on Apple Fruit

Three fitness parameters, i.e., virulence, sporulation, and stability of sensitivity phenotypes, were evaluated on detached apples in the absence and presence of fungicide selection pressure during prolonged cold storage to mimic conditions typically found in commercial packinghouses. Fitness parameters were evaluated in ten *P. expansum* isolates, i.e., two isolates representing each phenotype (Table 1).

Fuji apples harvested at commercial maturity in October 2022 and 2023 were sterilized and prepared for inoculation as described previously [43]. For trials without selection pressure, punctured apples (two punctures 4 mm wide and 4 mm deep per fruit) were inoculated with 20 µL of spore suspensions at 10^5^ spores mL^−1^. To assess the virulence and sporulation under selection pressure, punctured fruits were dipped in suspensions of Mertect 340F (TBZ), Scholar SC (FDL), or Shield-Brite Penbotec 400 SC (PYR) at the label rate of 1.25 mL L^−1^ for 1 min, allowed to dry for 4 h, and then inoculated with 20 µL of spore suspensions at 10^5^ spores mL^−1^. For each isolate, inoculation method, and year, four replicates, consisting of three apples each, were used. Inoculated apples were placed in clean plastic clamshells, each holding 12 fruits and stored at 1.5 °C. Blue mold incidence was monitored, and lesion diameters were measured in two perpendicular directions monthly up to 6 months. Sporulation was assessed after 6 months by resuspending the spores collected from the lesions of each fruit in 20 mL of sterile distilled water containing 0.01% Tween 20. The suspensions were filtered through cheesecloth, diluted appropriately, and used to enumerate spores in triplicate using a hemacytometer.

The stability of sensitivity phenotypes in vivo was assessed with and without selection pressure conditions on Fuji apples prepared and treated as described above. For each selection pressure condition, three apples with two inoculated punctures each were used for each isolate in 12-fruit clamshells, and the fruits were stored at 1.5 °C in a regular atmosphere. Every 60 days, a plug was dissected from the inner side of the decayed lesion and used to inoculate surface sterilized and punctured apples. This process was repeated four times, using apples that had been punctured and treated with TBZ, PYR, and FDL at 1.25 mL L^−1^, and a control treatment with water. At each transfer, the diameter of the lesions was measured. At the fourth and final transfer (eight months; G_m_8), spores were collected from the lesions using a sterile cotton swab. The sensitivity of the G_m_8 isolates to TBZ, PYR, and FDL was evaluated using the discriminatory doses used above.

### 2.4. Expression Analysis of Fitness-Related Genes in P. expansum Isolates

Seven fitness-related target genes were selected, i.e., *Ac1* (PEX2_003910), *Blistering1* (PEX2_008940), *BrlA* (PEX2_076900), *LaeA* (PEX2_005650), *OS-2* (PEX2_061660), *RacA* (PEX2_19970), and *VeA* (PEX2_043190). These genes have been documented to influence various fitness parameters examined in this study. To measure gene expression relative to two reference genes, *28s* (NG_069649) and *CaM* (DQ911134), the 15 *P. expansum* isolates were grown either on PDA without elicitation or with elicitation on PDA amended with 0.13, 0.01, and 0.01 µg mL^−1^ of TBZ, PYR, and FDL, equivalent of 1/75, 1/50, and 1/50 of their respective discriminatory doses, established as the highest doses allowing growth of wild-type isolates in preliminary experiments. The plates were inoculated with 10 µL of spore suspensions at 10^5^ conidia mL^−1^ of each isolate and incubated for 7 days at 20 °C.

Total RNA was extracted from mycelia and conidia harvested from the 7-day-old PDA cultures using *Quick*-RNA Fungal/Bacterial Miniprep ^TM^ kit (Zymo Research, Irvine, CA, USA) following the manufacturer’s protocol. The RNA was suspended in nuclease-free water (VWR, Radnor, PA, USA) and residual genomic DNA was eliminated through two rounds of RQ1 RNase-Free DNase treatment (Promega, Madison, WI, USA). The 260/280 ratio of the RNA samples was determined by nanodrop to be between 1.94 and 2.3. First-strand cDNA synthesis was performed using a qScript cDNA synthesis kit (Quantabio, Beverly, MA, USA) and stored in nuclease-free water at −20 °C. Quantitative PCR (qPCR) reactions were performed in a Bio-Rad CFX96 ^TM^ Real-Time PCR detection system (Bio-Rad Inc., Hercules, CA, USA). The reaction mixtures consisted of 10 µL of PerfeCTa SYBR Green FastMix (Quantabio, Beverly, MA, USA), 5 µL of cDNA template at 0.6 ng µL^−1^, primers (Table 2), and nuclease-free water (VWR, Radnor, PA, USA) to a final reaction volume of 20 µL.

The recommended thermal cycling protocol for PerfeCTa SYBR Green FastMix was used with an annealing temperature of 60 °C, except for *Ac1*, *LaeA*, and *BrlA* (Supplementary Table S1). Reaction conditions were optimized independently for each gene to achieve efficiencies of 90 to 110%. CFX Maestro ^TM^ software version 2.1 was used to analyze qPCR data. Relative gene expression was calculated using the 2^−ΔΔCt^ method, as described previously [44,45]. The expression data presented herein are averages of three biological replicates with three technical replicates per gene and treatment combination. The “sample maximization” experimental set-up for multi-plate qPCR studies [45] was used to minimize technical variation between samples.

### 2.5. Data Analysis

Data from repeat trials were combined for statistical analysis for all fitness parameters studied except conidial germination at 1.5 °C on intermediate media as well as virulence and sporulation on detached Fuji, which were analyzed by year. Normal distribution of the data was assessed using the Shapiro–Wilk test. All normal datasets were analyzed in R using type I ANOVA followed by Tukey’s post hoc test (*p* = 0.05). Relative gene expression values were box–cox-transformed and, if normalized, were analyzed via type II ANOVA followed by Tukey’s post hoc test to assess the interaction between the treatment (elicitation vs. non-elicitation) and isolate effects. For non-normal datasets, Kruskal–Wallis followed by Dunn’s test with Benjamini–Hochberg *p*-value adjustment was used (*p* = 0.05). A fitness change was calculated for the resistant isolates relative to the average of the three wild-type isolates for each fitness parameter. A fitness change (Fc) was calculated using the formula F_C_ = (X_R_ − X_WT_)/((X_R_ − X_WT_)/2) × 100 [46], where F_C_ = fitness change (%), X_R_ = the mean value of the given parameter in the resistant isolate, and X_WT_ = the mean value of the three wild-type isolates for the same parameter. A cumulative fitness change (CF_C_) was then expressed as the sum of the percentage of gain or loss across all parameters for each isolate.

## 3. Results

### 3.1. Comparison of Fitness Capabilities of P. expansum Isolates In Vitro

After 24 h incubation at 20 °C, no significant differences (*p* > 0.05) in germination rates were observed among isolates on IM, with all isolates germinating at rates ≥99.4% (Table 3) regardless of their phenotype, which were comparable to the germination rates on PDA (Supplementary Table S2). Germination was low in all isolates after 24 h on 2% WA at 20 °C, whereas after 48 h, all resistant isolates, except the dual-resistant TBZ^R^PYR^R^ isolate Pe-08 and PYR^R^FDL^R^ isolates Pe-153 and Pe-2501, displayed significantly (*p* < 0.05) lower germination rates (<80%) compared to the wild-type isolates (Table 3). At 1.5 °C, germination on IM and PDA was only observed after 48 h with the dual-resistant TBZ^R^PYR^R^ isolates exhibiting the lowest germination rates (1.2 to 25.9%), in contrast to the dual-resistant PYR^R^FDL^R^ isolates, which had the greatest germination rates ranging from 30.8 to 51.8% (Table 4). Germination on WA at 1.5 °C was absent up to 96 h. After 10 days of incubation, the resistant isolates, except for the single TBZ^R^ isolate Pe-23 and PYR^R^FDL^R^ isolate Pe-2501, exhibited a significant reduction in germination compared to the wild-type isolates (Table 4).

Most isolates grew faster on IM compared to PDA at both 20 and 1.5 °C (Table 3 and Table 4). After 15 days at 20 °C, all isolates grew similarly on IM regardless of their sensitivity phenotype (Table 3) with no differences seen between isolates at 5 and 10 days (Supplementary Table S2). On PDA, the growth of the TBZ^R^ isolates Pe-184 and Pe-219 was significantly greater than that of the wild-type isolates (*p* < 0.05), but no difference in growth was observed among the 10 other resistant isolates (Table 3). There were no significant growth differences between resistant isolates and the three wild-type isolates on IM or PDA after 120 days of incubation at 1.5 °C (Table 4) as well as 30, 60, and 90 days (Supplementary Table S2). Sporulation was not affected by the isolates regardless of their sensitivity phenotype after 15 days at 20 °C or after 120 days at 1.5 °C, regardless of the growth medium (Table 3 and Table 4).

Sensitivities to OSS and ROS were assessed on PDA amended with 12% NaCl or 30 mM of methyl viologen, respectively. After 15 days at 20 °C, the three single TBZ^R^, two dual TBZ^R^PYR^R^ isolates, and one PYR^R^FDL^R^ isolate grew significantly (*p* < 0.05) faster than the wild-type isolates on PDA amended with 12% NaCl (Table 3). At 1.5 °C, the triple-resistant isolates showed significantly reduced growth than the wild-type isolates after 120 days on PDA amended with 12% NaCl (Table 4). Moreover, increased sensitivity to ROS was observed in some dual- and triple-resistant isolates compared to the single TBZ^R^ and wild-type isolates after 15 days at 20 °C. Notably, two of the TBZ^R^PYR^R^FDL^R^-resistant isolates, Pe-2754 and Pe-3045, grew 6 and 16 mm, respectively, compared to >40 mm in the remaining isolates (Table 3). No growth was observed for any of the 15 isolates on PDA amended with 30 mM of methyl viologen after 120 days at 1.5 °C (Table 4).

The stability of the sensitivity phenotypes assessed after 10 monthly transfers on PDA at 1.5 °C showed no significant shifts in EC_50_ values for TBZ and FDL between G0 and G_m_10 in either the spore germination or mycelial growth inhibition assays, except for isolate Pe-2517, which showed an increase in EC_50_ for FDL based on the spore germination assay (Supplementary Table S2). In contrast, EC_50_ values for PYR increased by 4- to 25-fold, 1- to 5-fold, and 1- to 9-fold in TBZ^R^PYR^R^, PYR^R^FDL^R^, and TBZ^R^PYR^R^FDL^R^ isolates, respectively, according to the spore germination assay. However, increases in EC_50_ values were lower or minor based on the mycelial growth assay (Supplementary Table S3).

### 3.2. Virulence, Sporulartion, and Resistance Stability in P. expansum Isolates on Detached Apples

**Figure 1:**
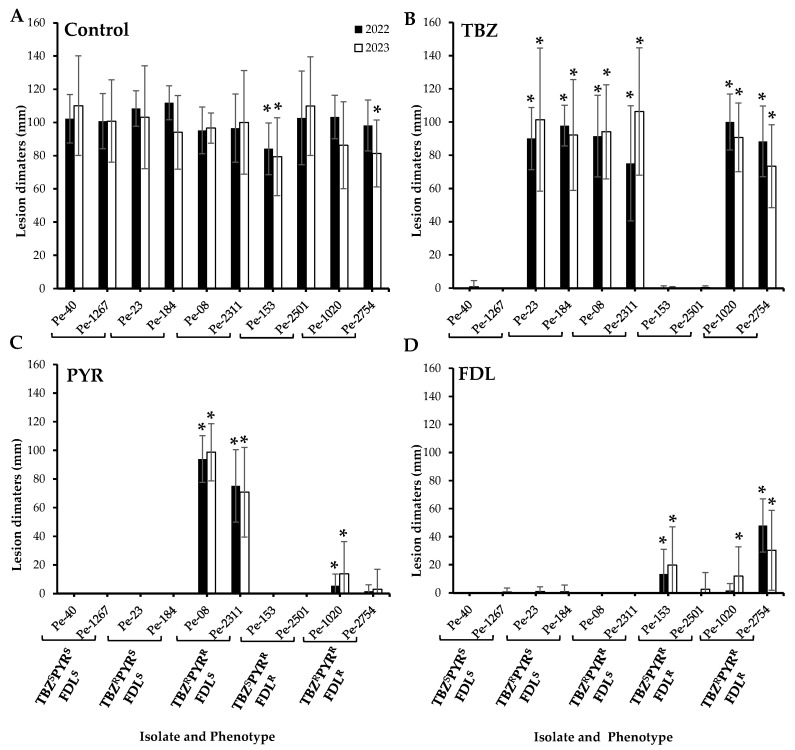
Virulence (lesion diameters in mm) of ten *Penicillium expansum* isolates on wounded untreated Fuji apples (control, (**A**)) and apples treated with thiabendazole (TBZ, (**B**)), pyrimethanil (PYR, (**C**)), and fludioxonil (FDL, (**D**)) at the label rates then inoculated with spore suspensions from each isolate. Lesion diameters were measured after six months at 1.5 °C. The 2022 and 2023 trials were analyzed separately. Error bars indicate the standard deviations of the means. Asterisks indicate statistical difference from the TBZ^S^PYR^S^FDL^S^ (wild-type) isolates based on Kruskal–Wallis and Dunn’s tests at *p* < 0.05. S and R denote sensitive and resistant isolates, respectively.

Regardless of their sensitivity phenotype, all ten tested *P. expansum* isolates caused 100% blue mold incidence on untreated Fuji apples at all inspection times (monthly between two to six months) at 1.5 °C, but some differences were observed in their virulence after six months of storage. For instance, the dual-resistant PYR^R^FDL^R^ isolate Pe-153 was significantly less virulent compared to the wild-type isolates in both the 2022 and 2023 trials, while the triple-resistant TBZ^R^PYR^R^FDL^R^ Pe-2754 isolate was less virulent only in the 2023 trial (*p* < 0.05) (Figure 1A). Under fungicide selection pressure, only isolates with corresponding resistance phenotypes were able to infect the Fuji apples after six months of cold storage (Figure 1A). There were no significant differences in the size of lesions caused by the isolates that were resistant to TBZ on TBZ-treated apples (Figure 1B). On PYR-treated apples, the dual-resistant TBZ^R^PYR^R^ isolates exhibited a significantly greater lesion diameter compared to the other isolates. The triple-resistant isolates Pe-1020 and Pe-2754 caused small lesions on PYR-treated apples (Figure 1C), but no lesions were observed on apples infected by the dual-resistant PYR^R^FDL^R^ isolates on PYR-treated apples in both years (Figure 1C). Overall, the FDL^R^ isolates caused smaller lesions on FDL-treated apples. Although, the dual-resistant PYR^R^FDL^R^ isolate Pe-2501 remained avirulent in 2022, both the PYR^R^FDL^R^ and the triple-resistant isolates produced lesions ranging from 1.7 to 48.0 mm in 2022 and 2.5 to 30.4 mm in 2023 on FDL-treated apples (Figure 1D).

**Figure 2:**
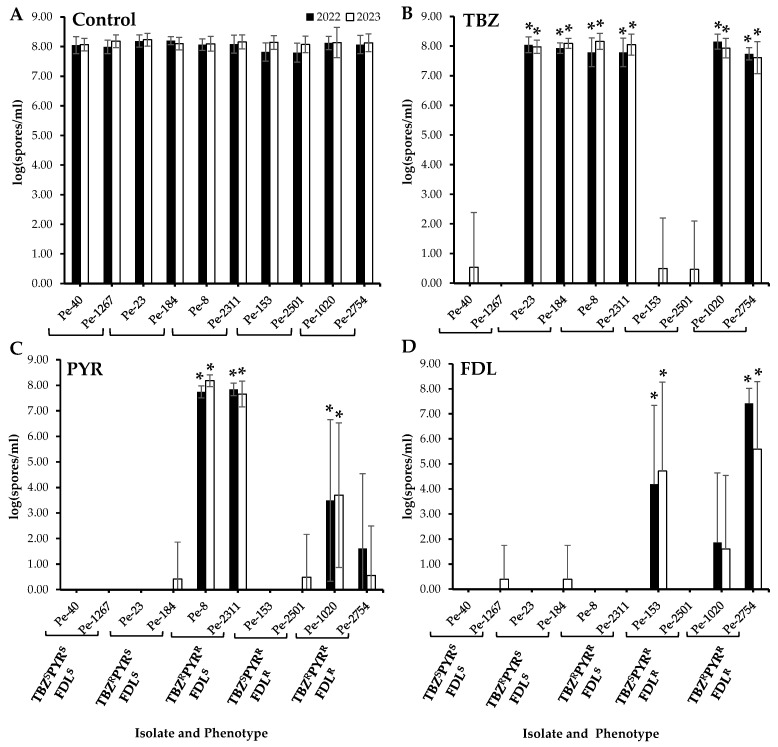
Sporulation (spores mL^−1^) of *Penicillium expansum* isolates with different sensitivity phenotypes on wounded untreated Fuji apples (control, (**A**)) and on apples treated with thiabendazole (TBZ, (**B**)), pyrimethanil (PYR, (**C**)), and fludioxonil (FDL, (**D**)) at the label rates then inoculated with spore suspensions from each isolate. Lesion diameters were measured after six months at 1.5 °C. Error bars indicate the standard deviations of the means. Asterisks indicate statistical difference from the TBZ^S^PYR^S^FDL^S^ (wild-type) isolates based on Kruskal–Wallis and Dunn’s tests at *p* < 0.05. S and R denote sensitive and resistant isolates, respectively.

No significant differences in sporulation were observed between the wild-type and the resistant isolates on untreated apples after six months of cold storage (Figure 2A). On TBZ-treated apples, the sporulation of all TBZ^R^ isolates, i.e., those with single, dual, and triple resistance, was significantly higher than that of the TBZ^S^ isolates (Figure 2B). On PYR-treated fruit, significantly increased sporulation (*p* < 0.05) was observed in the two TBZ^R^PYR^R^ isolates as well as the triple-resistant isolate Pe-1020 (Figure 2C), while only the PYR^R^FDL^R^ isolate Pe-153 and the triple-resistant isolate Pe-2754 exhibited significantly increased spore production (*p* < 0.05) on FDL-treated Fuji apples (Figure 2D).

Consistent with the in vitro findings, no significant changes in phenotypic sensitivity for TBZ and FDL were observed after eight months (four transfers at two-month intervals) on detached Fuji apples at 1.5 °C, based on the spore germination assay and discriminatory doses used for isolate characterization. For PYR, isolate Pe-153 exhibited reduced sensitivity after four transfers on apples.

### 3.3. Cumulative Phenotypic Fitness Changes in P. expansum-Resistant Isolates

A cumulative fitness change (CF_C_) expressed as the sum of the percentage of gain or loss across all parameters in the resistant isolates relative to the wild-type isolates was calculated for each isolate and phenotype. All resistant isolates, regardless of their sensitivity phenotypes, showed a cumulative fitness loss on WA with mean cumulative fitness changes (CF_C_) ranging from −72 to −86% and −15 to −26% at 1.5 and 20 °C, respectively, with no major difference among the phenotypes (Table 5). On the IM at 1.5 °C, the dual-resistant PYR^R^FDL^R^ isolates exhibited a mean fitness gain of 36%, to the contrary of the TBZ^R^, TBZ^R^PYR^R^, and TBZ^R^PYR^R^FDL^R^ phenotypes with respective mean fitness losses of −70, −103, and −8%. At 20 °C, only minor gains or losses were observed on IM. On the nutrient-rich medium PDA, all fungicide sensitivity phenotypes registered fitness losses at 1.5 °C, whereas fitness loss was only observed in PYR^R^FDL^R^ and the TBZ^R^PYR^R^FDL^R^ phenotypes at 20 °C. On detached untreated apples, the single TBZ^R^ isolates exhibited a fitness gain of 7%, while the three other phenotypes exhibited moderate fitness losses between −5 and −14% (Table 5). On fungicide-treated apples, large fitness gains were observed across most phenotypes, except for the TBZ^R^PYR^R^ phenotypes on FDL-treated apples (Table 5).

### 3.4. Gene Expression of Fitness-Related Genes in P. expansum Isolates with Different Fungicide Sensitivity Phenotypes

**Figure 3:**
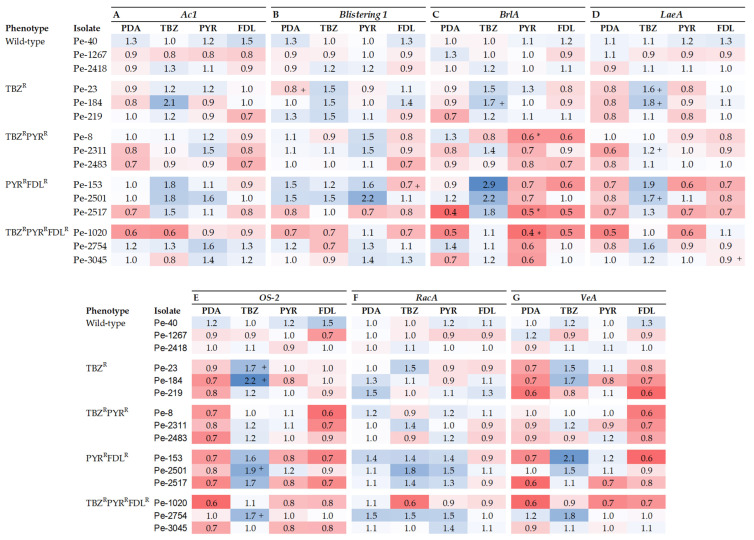
Heatmap representing the expression of *Ac1* (**A**), *Blistering1* (**B**), *OS*-2 (**C**), *RacA* (**D**), *BrlA* (**E**), *LaeA* (**F**), and *VeA* (**G**) genes relative to the reference genes 28s and CMD in *Penicillium expansum* isolates with different sensitivity phenotypes after 7 days at 20 °C on PDA (control) and on PDA amended with thiabendazole (TBZ), pyrimethanil (PYR), and fludioxonil (FDL) at 0.13, 0.01, and 0.01 µg mL^−1^, respectively. Data bars are the means of nine values from three biological replicates. Error bars indicate the standard deviations of the means. S and R denote sensitive and resistant isolates, respectively. Pluses indicate statistical difference between isolates as a factor of fungicide elicitation compared to the control. Asterisks indicate statistical differences in resistant isolates from the wild-type isolates; + indicates statistical differences in elicitated samples compared to the PDA treatment alone. (**A**–**G**) and (**E**,**F**) were analyzed with type II ANOVA/Tukey-HSD and type I ANOVA/Tukey-HSD or Kruskal–Wallis and Dunn’s tests, respectively, at *p* ≤ 0.05.

The relative expression (RE) of seven fitness-related genes was measured in vitro on PDA with and without elicitation via sub-lethal doses of TBZ, PYR, or FDL. The *Ac1* gene, which codes for adenylyl cyclase, is linked to germination, growth, and virulence in fungi. In a cross-isolate comparison, *Ac1* was significantly up-regulated (2-fold) in the TBZ^R^ isolate Pe-184 by TBZ elicitation compared to the control (Figure 3A). *Ac1* expression was significantly (*p* < 0.05) down- and up-regulated in the TBZ^R^PYR^R^FDL^R^ and TBZ^R^ phenotypes, respectively, whereas its expression was up-regulated by PYR in the TBZ^R^PY^R^ phenotype (Supplementary Figure S1A). Likewise, the expression of the *Blistering1* gene, linked to growth, virulence, and patulin production, was largely unaffected in most isolates except for the TBZ^R^ isolate Pe-23 and the PYR^R^FDL^R^ isolate Pe-153, which were, respectively, up- and down-regulated (*p* < 0.05) by TBZ and FDL compared to the control (Figure 3B). A sensitivity phenotype effect (*p* < 0.05) was observed in the TBZ^R^ phenotype where expression was up-regulated by TBZ (Supplementary Figure S1B).

The expression of the *OS-2* gene, implicated in osmotic stress tolerance, was significantly up-regulated by TBZ in four isolates, Pe-23 and Pe-184 (TBZ^R^), Pe-2483 (TBZ^R^PYR^R^), and Pe-2501 (PYR^R^FDL^R^), across three phenotypes (Figure 3C). The expression of *OS-2* was down-regulated by FDL in the TBZ^R^PYR^R^ phenotype and was up-regulated by PYR in the TBZ^R^PYR^R^ phenotype and by TBZ in all resistant phenotypes (Figure S1C). Moreover, the RE of the *RacA* gene, linked to oxidative stress response and patulin production, was significantly down-regulated (*p* < 0.05) by TBZ in the TBZ^R^ isolate Pe-219 and the TBZ^R^PYR^R^FDL^R^ isolate Pe-1020 (Figure 3D), whereas no differences were observed between phenotypes (Figure S1D).

The RE of *BrlA,* a transcription factor important for sporulation in *P. expansum*, was significantly down-regulated by PYR in isolates Pe-8 (TBZ^R^PYR^R^), Pe-2517 (PYR^R^FDL^R^), and Pe-1020 (TBZ^R^PYR^R^FDL^R^) compared to the sensitive isolates (Figure 3E). There was also a significant phenotype effect as *BrlA* RE was down-regulated (*p* < 0.05) in the TBZ^R^PYR^R^, PYR^R^FDL^R^, and TBZ^R^PYR^R^FDL^R^ phenotypes compared to the sensitive phenotype (Figure S1E). *BrlA expression* was significantly up-regulated (*p* < 0.05) in the TBZ^R^ and PYR^R^FDL^R^ phenotypes by TBZ elicitation compared to the control (Figure S1E). *LaeA* is another transcription factor linked to virulence, sporulation, and patulin production. A cross-isolate comparison showed *LaeA* to be up-regulated by TBZ in isolates Pe-23 and Pe-184 (TBZ^R^), Pe-2311 (TBZ^R^PYR^R^), and Pe-2501 (PYR^R^FDL^R^), whereas the gene was over-expressed (*p* < 0.05) by FDL in the triple-resistant isolate Pe-1020 compared to the non-elicited control (Figure 3F). A comparison between phenotypes showed that *LaeA* was down-regulated in all non-elicited resistant phenotypes. PYR down-regulated *LaeA* expression (*p* < 0.05) in the PYR^R^FDL^R^ and TBZ^R^PYR^R^FDL^R^ phenotypes, while expression was down-regulated by FDL in the PYR^R^FDL^R^ phenotype compared to the sensitive phenotype (Supplementary Figure S1F).

The last gene investigated was *VeA*, reported to influence growth, virulence, sporulation, and patulin production. Its expression was very similar among the 15 *P. expansum* isolates except in the TBZ^R^ isolates Pe-23 and Pe-184, which showed significantly increased expression (*p* < 0.05) compared to the wild-type isolates (Figure 3G). *VeA* expression among phenotypes was down-regulated in TBZ^R^, PYR^R^FDL^R^, and TBZ^R^PYR^R^FDL^R^ in non-elicited treatment as well as by FDL elicitation in the TBZ^R^, TBZ^R^PYR^R^, and PYR^R^FDL^R^ phenotypes compared to the sensitive phenotype (Figure S1G). In contrast, TBZ elicitation up-regulated *VeA* RE significantly in the TBZ^R^ and PYR^R^FDL^R^ phenotypes.

## 4. Discussion

*P. expansum* isolates with varying fungicide sensitivity phenotypes, i.e., isolates sensitive to TBZ, PYR, and FDL or resistant to one, two, or all three fungicides, were collected from commercial packinghouses in the U.S. PNW states of WA and OR. Their germination, growth, sporulation, response to osmotic and oxidative stress, and phenotype stability were assessed in vitro, while virulence, sporulation, and stability were evaluated on detached apples under different temperatures and fungicide selection pressures. Resistant isolates exhibited significantly reduced conidial germination on a nutrient-deficient medium (WA) at both 20 and 1.5 °C compared to the wild-type isolates. However, this reduction was pronounced on nutrient-rich media like PDA and IM, which simulates plant surface nutrient availability [38]. While a correlation between fungicide resistance and reduced germination has been documented in *Aspergillus parasiticus* [47], only one previous study using *P. expansum* lab mutants found no effect of resistance to PYR and FDL on germination on PDA after 24 h [31]. Our study is the first to provide more insights into such a correlation in wild-type and field-resistant isolates. Given that germination is crucial for fungal infection, impaired germination may hinder the successful establishment of resistant isolates. These findings suggest that *P. expansum* isolates resistant to TBZ, PYR, and/or FDL may pose lower disease risk compared to wild-type isolates in cold and nutrient-deficient environments such as bins, storage rooms, and flume water, provided that organic matter sources (e.g., remaining decayed fruit) are absent. This highlights the importance of thoroughly cleaning and sanitizing postharvest facilities and equipment to minimize the risk of decay by fungicide-resistant isolates.

In contrast with spore germination, the growth of *P. expansum* isolates on three agar media was not affected by their fungicide sensitivity phenotype, either after 120 days at 1.5 °C or after 15 days at 20 °C. Previous studies have reported mixed findings regarding growth differences among isolates with varying resistance profiles. For instance, lab mutants and field *P. expansum* isolates resistant to TBZ exhibited no growth differences on PDA after 7 days at 20 °C but displayed higher severity than the sensitive isolates on detached pears and apples [12,13]. Conversely, *P. expansum* lab mutants resistant to TBZ, PYR, or/and FDL exhibited variable growth on PDA after one week and 10 weeks at 20 and 0 °C, respectively [31]. Specifically, lab mutants resistant to FDL, PYR, and dual-resistant to TBZ and FDL showed reduced growth compared to the parental isolates, whereas the triple-resistant isolates grew at rates comparable to the wild-type. Similarly, Greek field isolates with single resistance to cyprodinil (FRAC 9) exhibited similar growth on PDA and virulence on Red Delicious apples compared to the wild-type isolates, whereas isolates with triple resistance to FDL, iprodione (FRAC 2), and tebuconazole (FRAC3) exhibited reduced growth on PDA and reduced virulence after 7 days at 20 °C [22]. Discrepancies between studies may stem from the use of lab mutants, which often display reduced fitness, as well as differences in resistance phenotypes investigated, experimental methods, or environmental conditions. Nevertheless, when considering both the current findings and previous research on various fitness parameters, *P. expansum* isolates resistant to one or more fungicide appear capable of maintaining adequate growth across a range of nutrient environments, suggesting that resistance does not necessarily compromise growth capacity under the tested conditions.

The ability of plant pathogens to withstand external stressors, such as osmotic (OSS) and oxidative (ROS) stress, plays a critical role in their survival under adverse conditions and their potential to cause disease. Fludioxonil is believed to disrupt the high-osmolarity glycerol (HOG) pathway, which regulates glycerol synthesis, an essential osmoregulatory mechanism [18,26]. As a result, the sensitivity to OSS is commonly evaluated in FDL-resistant fungal pathogens. In this study, the fungicide resistance phenotype of *P. expansum* field isolates did not significantly affect their OSS response after 15 days at 20 °C. However, dual PYR^R^FDL^R^- and triple TBZ^R^PYR^R^FDL^R^-resistant isolates showed significantly reduced growth on PDA amended with 12% NaCl at 1.5 °C after 120 days. These findings align with previous reports of increased OSS sensitivity in FDL^R^ isolates of *P. expansum* and *B. cinerea* [31,48] but not in *P. digitatum* [18]. The difference observed in OSS sensitivity between *P. expansum* and *P. digitatum* could be explained by the relatively higher dose of NaCL_2_ used in our study compared to that used by Kanetis et al. [18]. Furthermore, ROS plays a crucial role in fungal infection, signaling, and pathogenicity [49]. While ROS sensitivity did not differ significantly among wild-type and resistant isolates, the dual PYR^R^FDL^R^ isolates Pe-2501 and Pe-2517, as well as the triple-resistant isolates Pe-2754 and Pe-3045, exhibited the lowest growth among the isolates after 15 days on PDA amended with 30 mM of methyl viologen. Although ROS sensitivity in *P. expansum* has not been extensively studied, its link to the HOG pathway has been demonstrated in FDL-resistant strains of *B. cinerea* and *Saccharomyces cerevisiae* [50,51]. In contrast, the deletion of *Hog1*, a key kinase in the HOG pathway, did not affect ROS sensitivity in *P. digitatum*. The heightened OSS and ROS sensitivity in FDL^R^ isolates may explain their low prevalence in PNW packinghouses after nearly 20 years of FDL use. Notably, no substantial shift in EC_50_ to FDL, TBZ, or PYR among the resistant isolates occurred after ten weekly transfers, suggesting that unstable resistance mechanisms are unlikely to explain their limited occurrence. Nevertheless, continued monitoring and further investigation into stress response mechanisms are essential to mitigate the risk of sudden increase in resistance.

Understanding the pathogenic fitness of fungicide-resistant populations is important for developing management strategies. In 80% of *P. expansum* isolates inoculated on fungicide-untreated Fuji apples, no significant differences in lesion size were observed after 140 days at 1.5 °C, even if some dual PYR^R^FDL^R^- and triple TBZ^R^PYR^R^FDL^R^-resistant isolates produced smaller lesions compared to the wild-type isolates after six months of cold storage. Previous studies reported that TBZ^R^ *P. expansum* isolates from Italy and Greece were more virulent than the wild-type isolates [12,13]. In this study, TBZ^R^ isolates behaved differently across years with larger lesions than the wild-type isolates in 2022, but not in 2023. Previously, laboratory PYR^R^ mutants showed virulence comparable to their parental isolates, but FDL^R^ and TBZ^R^FDL^R^ mutants exhibited temperature-dependent virulence, which was reduced at 0 °C but not at 20 °C [31]. Field isolates resistant to FDL and other preharvest fungicides, not labeled for blue mold management, have also demonstrated reduced virulence in short-term storage at 20 °C [23,24]. Despite some variation in virulence, all resistant phenotypes, i.e., single, dual, and triple, could infect apples, produce substantial lesions, and sporule abundantly under selection pressure. Moreover, these isolates maintained stable resistance over 10 months in vitro and eight months on fruits. These findings align with previous reports [27,49] and reinforce concerns that continued fungicide use may accelerate the selection for additional resistant *P. expansum* populations in commercial packinghouses.

Comparing individual fitness parameters within and across studies to assess the overall impact of fungicide resistance on fitness is inherently challenging due to methodological and biological variability. In natural environments, however, fitness costs likely result from the combined effect of multiple components, such as germination, growth, sporulation, stress tolerance, and virulence, rather than from a single trait. To better capture these cumulative effects, we calculated a cumulative fitness cost change (CFc), representing the overall gain or loss in fitness of resistant relative to the wild-type isolates. This analysis revealed clear overall fitness reductions in the resistant phenotypes under most in vitro conditions, especially under cold storage, compared to the wild-type isolates. These reductions were not always apparent when examining individual fitness components separately. Furthermore, the CFc analysis highlighted a negative impact of prolonged selection pressure on *P. expansum* virulence on apples. The CFc approach corresponds with the observed low prevalence of dual- and triple-resistant phenotypes, especially those resistant to FDL, in PNW packinghouses, suggesting that cumulative fitness costs, rather than isolated trait penalties, may limit their persistence. While further mathematical and statistical refinement is needed, CFc offers a promising framework for estimating fitness costs in plant pathogens under natural conditions.

To investigate whether resistance to a single or multiple fungicides affects the expression and functionality of genes associated with key fitness traits, the expression of seven fitness-related genes was evaluated in the absence and presence of fungicide selection pressure. Exposure to TBZ significantly elevated the expression of *LaeA* and *OS2*, which play roles in virulence, sporulation, and sensitivity to osmotic stress (OSS), across all resistant phenotypes, as well as increased expression of *Ac1*, *Blistering1*, *BrlA*, and *VeA* in select phenotypes. While differences in OSS sensitivity were observed between isolates and phenotypes in vitro, the differential expression of certain genes may help explain the absence of fitness penalties, such as in growth, virulence, or sporulation, both in vitro and on detached fruit. Previous research reported differential gene expressions due to fungicide application, particularly in resistant isolates [52]. Notably, this gene expression induction was observed only with TBZ. In contrast, PYR treatment elicited only minor changes in expression at the phenotype level and no discernible impact at the isolate level. FDL treatment had no observable effect on gene expression at the phenotype level and only a minor impact at the isolate level. This lack of response is particularly surprising for *OS-2*, given the well-established role of the HOG pathway in mediating fludioxonil responses across several fungal species [9,18,26,29]. Fludioxonil is thought to stimulate polyol (e.g., glycerol) production via the HOG pathway and *OS-2* in response to osmotic stress; however, this mechanism may differ in *P. expansum*, as prior studies have not linked *OS-2* expression to osmotic stress in this species [38] nor were mutations detected in OS genes of *P. expansum* and other *Penicillium* spp. [9].

Comparisons between PYR-resistant and -sensitive phenotypes revealed consistently lower expression of *BrlA* in resistant isolates treated with PYR or FDL. *BrlA*, a C2H2-type zinc-finger transcription factor, is essential for conidiophore development and sporulation [35]. Additional differences in *LaeA* and *VeA* expression were observed between resistant and sensitive phenotypes; however, no consistent differences emerged at the individual isolate level. These results did not align with in vitro and in vivo sporulation studies, which showed no significant differences between isolates or phenotypes. This suggests that assessing fitness penalties at the molecular level, by measuring the expression of fitness-related genes, may be more effective in detecting potential fitness costs associated with fungicide resistance development. Although the precise resistance mechanisms to PYR and FDL in *P. expansum* remain unclear, if resistance involves increased metabolic demand, fungicide exposure may redirect energy toward detoxification or efflux pump activity at the expense of reproductive functions, leading to reduced expression of genes such as *BrlA* [53]. The relative expression analysis in this study highlights the complex nature of fungal fitness. Numerous transcription factors and regulatory pathways influence the life cycle and virulence of *P. expansum*, underscoring the need for broader approaches, such as RNA sequencing, to investigate both known and novel fitness-related genes.

## 5. Conclusions

Our study established variable associations between fungicide resistance and fitness in *P. expansum*. We demonstrated that under low-nutrient conditions, the germination capacity of fungicide-resistant isolates is significantly reduced, and that dual- and triple-resistant isolates may be more vulnerable to osmotic and oxidative stresses. Furthermore, we developed a cumulative fitness cost (CFc) metric that revealed reduced overall fitness in resistant phenotypes. However, no association was found between fungicide resistance and virulence, suggesting that resistant phenotypes may persist and cause a greater risk in commercial packinghouses.

## Figures and Tables

**Table 1:** Characteristics of *Penicillium expansum* isolates tested in this study.

	Sensitivity	Sensitivity	Year			Geographic Origin		Tested ^c^	
Isolate ID	Phenotype ^a^	Group	Isolated	Host	Cultivar	State ^b^	County	In Vitro	In Vivo
*Pe*-40	TBZ^S^PYR^S^FDL^S^	Sensitive to all	2016	Apple	Fuji	WA	Franklin	+	+
*Pe*-1267			2017	Apple	Fuji	WA	Grant	+	+
*Pe*-2418			2017	Apple	Gala	WA	Grant	+	−
*Pe*-23	TBZ^R^	Single-Resistant	2016	Apple	Gala	WA	Okanogan	+	+
*Pe*-184			2018	Pear	Bosc	OR	Hood River	+	+
*Pe*-219			2017	Apple	Red Delicious	WA	Yakima	+	−
*Pe*-08	TBZ^R^PYR^R^	Dual-Resistant	2016	Apple	Gala	WA	Okanogan	+	+
*Pe*-2311			2016	Apple	Fuji	WA	Grant	+	+
*Pe*-2483			2016	Apple	Gala	WA	Grant	+	−
*Pe*-153	PYR^R^FDL^R^		2016	Apple	Gala	WA	Okanogan	+	+
*Pe*-2501			2016	Apple	Gala	WA	Douglas	+	+
*Pe*-2517			2017	Apple	Gala	WA	Okanogan	+	−
*Pe*-1020	TBZ^R^PYR^R^FDL^R^	Triple-Resistant	2017	Apple	Gala	WA	Okanogan	+	+
*Pe*-2754			2017	Apple	Gala	WA	Benton	+	+
*Pe*-3045			2017	Apple	Granny Smith	WA	Yakima	+	−

^a^ TBZ, PYR, and FDL denote thiabendazole, pyrimethanil, and fludioxonil, respectively. S and R depict sensitive and resistant isolates to specific fungicide, respectively. ^b^ WA and OR indicate Washington and Oregon states, respectively. ^c^ + or − denote isolate tested or not, respectively, in vitro and/or in vivo.

**Table 2:** Fitness-related genes and primers used in RT-qPCR relative expression analysis.

Gene	Gene ID	Hypothesized Fitness Parameter	Primer Name	Sequence (5′-3′)	Reference
*Ac1*	*PEX2_003910*	Growth/Germination/Virulence	PeAc1-rt-F2	AGTCACCCGTATCGAGGTATGC	This study
			PeAc1-rt-R2	CTCGGTGATGGGTACAGCGT	
*Blistering1*	*PEX2_008940*	Growth/Virulence/Patulin	R19_6445F	CACGGTCTTGCCACTTGCTCGT	[34]
			R19_6445R	TCGTTTCGAGTACGTCGCGCTG	
*BrlA*	*PEX2_076900*	Sporulation	dBrlA_F	CCTCGATGCCTCAATACA	[35]
			dBrlA_R	GTAAAGATTGGACGAGACAAG	
*LaeA*	*PEX2_005650*	Virulence/Sporulation/Patulin	PeLaeA-rt-F2	TCCGATCGCAAGATACCCGA	This study
			PeLaeA-rt-R2	GTCACACGGAAGCGGGTAGA	
*OS-2*	*PEX2_061660*	Sensitivity to Osmotic Stress	PeOS-2-rt-F2	CTGTAGCGAGAACACCCTCCG	This study
			PeOS-2-rt-R2	CGAGCAAGTCAACCGCATCG	
*RacA*	*PEX2_019970*	Sensitivity to ROS/Patulin	PeRacA-rt-F1	AACGTCAAAGCGAAGTGGTTC	This study
			PeRacA-rt-R1	CACGGTCGTCCCTCAGATCG	
*VeA*	*PEX2_043190*	Growth/Virulence/Sporulation/Patulin	PeVeA-rt-F2	CGGGAGCCAAGTCATCTGCT	This study
			PeVeA-rt-R2	GCATCGTTAGCCGGATCGGA	
*28S*	*NG_069649*	Reference	28SF	GGAACGGGACGTCATAGAGG	[32]
			28SR	AGAGCTGCATTCCCAAACAAC	
*CaM*	*DQ911134*	Reference	PeCMD-rt-F3	CTCACCATGATGGCTCGTAAGA	[39]
			PeCMD-rt-R3	GCGGAAATGAAACCGTTGTT	

**Table 3:** Germination, mycelial growth, sporulation, and sensitivity to exogenic stresses of 15 *Penicillium expansum* isolates with different sensitivity phenotypes in vitro at 20 °C.

Sensitivity		Spore Germination ^a^				Mycelial Growth ^b^				Sporulation ^c^				Sensitivity to Exogenic Stress ^d^			
Phenotype	Isolate	IM		WA		PDA		IM		PDA		IM		OSS		ROS	
TBZ^S^PYR^S^FDL^S^	*Pe*-40	99.9 ± 0.2	a ^ef^	88.1 ± 5.1	ab	38.6 ± 4.2	cde	45.3 ± 2.1	cde	8.04 ± 0.2	ab	7.72 ± 0.2	a	5.4 ± 2.5	de	42.0 ± 6.2	bcd
Wild-Type	*Pe*-1267	99.8 ± 0.4	a	92.7 ± 4.1	a	34.4 ± 4.8	e	44.8 ± 2.8	de	7.64 ± 0.5	b	7.73 ± 0.3	a	6.5 ± 0.6	de	27.9 ± 7.1	ef
	*Pe*-2418	99.8 ± 0.4	a	92.8 ± 3.6	a	40.6 ± 2.8	c	46.5 ± 1.9	abcde	8.19 ± 0.1	a	7.58 ± 0.3	a	6.4 ± 0.5	de	43.6 ± 4.1	bcd
	**Mean**	**99.9 ± 0.3**		**91.2 ± 4.8**		**37.9 ± 4.8**		**45.5 ± 2.4**		**7.96 ± 0.4**		**7.68 ± 0.2**		**6.1 ± 1.6**		**37.8 ± 9.2**	
TBZ^R^PYR^S^FDL^S^	*Pe*-23	99.7 ± 0.6	a	68.8 ± 9.4	fg	39.9 ± 1.8	cd	47.4 ± 3.1	abcde	7.89 ± 0.3	ab	7.71 ± 0.1	a	7.6 ± 1.1	bc	45.0 ± 3.1	b
Single-Resistant	*Pe*-184	99.7 ± 1.0	a	74.7 ± 6.9	def	45.1 ± 3.1	ab	45.9 ± 3.8	bcde	7.90 ± 0.3	ab	7.64 ± 0.2	a	8.4 ± 1.1	ab	47.0 ± 4.0	ab
	*Pe*-219	99.4 ± 0.8	a	80.1 ± 8.1	cde	47.1 ± 4.9	a	45.8 ± 3.5	bcde	8.01 ± 0.1	ab	7.60 ± 0.2	a	7.9 ± 0.5	ab	51.8 ± 3.8	a
	**Mean**	**99.6 ± 0.8**		**74.5 ± 9.3**		**44.1 ± 4.6**		**46.4 ± 3.5**		**7.96 ± 0.4**		**7.65 ± 0.2**		**8.0 ± 1.0**		**47.9 ± 4.6**	
TBZ^R^PYR^R^FDL^S^	*Pe*-08	99.9 ± 0.3	a	85.8 ± 6.6	abc	39.1 ± 4.5	cd	48.5 ± 2.2	ab	7.75 ± 0.2	b	7.53 ± 0.2	a	8.8 ± 0.7	a	37.2 ± 7.5	cde
Dual-Resistant	*Pe*-2311	99.9 ± 0.3	a	65.9 ± 5.7	g	40.7 ± 3.5	c	48.0 ± 1.8	abc	8.07 ± 0.1	ab	7.61 ± 0.2	a	7.4 ± 1.0	bc	41.8 ± 10.9	bcd
	*Pe*-2483	99.7 ± 0.6	a	72.5 ± 10.6	efg	41.1 ± 3.3	bc	47.8 ± 2.3	abcd	8.00 ± 0.1	ab	7.61 ± 0.2	a	4.5 ± 3.5	de	44.3 ± 3.4	bc
	**Mean**	**99.8 ± 0.4**		**74.7 ± 11.4**		**40.3 ± 3.8**		**48.1 ± 2.1**		**7.96 ± 0.4**		**7.58 ± 0.2**		**6.9 ± 2.8**		**41.1 ± 8.3**	
TBZ^S^PYR^R^FDL^R^	*Pe*-153	99.8 ± 0.4	a	81.7 ± 4.8	bcd	38.0 ± 4.1	cde	44.6 ± 2.6	e	7.73 ± 0.2	b	7.75 ± 0.2	a	3.6 ± 3.8	de	42.8 ± 11.1	bc
Dual-Resistant	*Pe*-2501	99.8 ± 0.5	a	81.8 ± 7.1	bcd	40.1 ± 2.8	cd	45.4 ± 2.9	cde	7.67 ± 0.4	b	7.73 ± 0.1	a	4.3 ± 2.5	e	22.6 ± 23.3	def
	*Pe*-2517	99.8 ± 0.4	a	72.3 ± 9.4	efg	39.1 ± 2.5	cd	45.0 ± 1.5	cde	7.90 ± 0.3	ab	7.64 ± 0.3	a	7.6 ± 0.5	bc	10.1 ± 19.7	ef
	**Mean**	**99.8 ± 0.5**		**78.6 ± 8.5**		**39.1 ± 3.3**		**45.0 ± 2.4**		**7.77 ± 0.3**		**7.71 ± 0.2**		**5.1 ± 3.1**		**25.2 ± 22.9**	
TBZ^R^PYR^R^FDL^R^	*Pe*-1020	99.9 ± 0.2	a	65.1 ± 11.1	fg	40.8 ± 4.7	c	49.2 ± 2.3	a	7.91 ± 0.1	ab	7.62 ± 0.3	a	6.9 ± 0.4	cd	42.2 ± 3.5	bcd
Triple-Resistant	*Pe*-2754	99.9 ± 0.2	a	72.6 ± 5.7	efg	36.0 ± 2.8	de	47.1 ± 3.0	abcde	7.98 ± 0.1	ab	7.66 ± 0.2	a	6.2 ± 0.7	de	16.3 ± 16.9	f
	*Pe*-3045	99.9 ± 0.3	a	74.2 ± 5.2	efg	41.5 ± 0.4	bc	47.3 ± 3.1	abcde	8.05 ± 0.1	ab	7.55 ± 0.0	a	4.7 ± 2.4	e	6.1 ± 17.7	f
	**Mean**	**99.9 ± 0.3**		**70.6 ± 8.7**		**39.4 ± 4.5**		**47.9 ± 2.9**		**7.98 ± 0.1**		**7.61 ± 0.2**		**5.9 ± 1.7**		**21.5 ± 21.0**	

^a^ Spore germination (%) on intermediate medium (IM) and water agar (WA) was assessed after 24 h and 48 h, respectively. ^b^ Mycelial growth (mm) was measured on PDA and IM after 15 d. ^c^ Sporulation (conidia/mL) was measured after 15 d on PDA and IM. ^d^ Sensitivity to exogenic stress (colony diameter in mm) was assessed after 15 days on PDA amended with 12% NaCl or with 30 mM of methyl viologen for osmotic stress (OSS) and reactive oxygen species (ROS), respectively. ^e^ Mean and standard deviation are shown for each isolate, medium, and fitness parameter combination. ^f^ Differences in letters indicate statistically significant difference between isolates based on ANOVA and Tukey-HSD (all parameters except germination) or Kruskal–Wallis and Dunn’s (germination) tests at *p* < 0.05.

**Table 4:** Germination, mycelial growth, sporulation, and sensitivity to exogenic stresses of 15 *Penicillium expansum* isolates with different sensitivity phenotypes in vitro at 1.5 °C.

Sensitivity		Spore Germination ^a^				Mycelial Growth ^b^				Sporulation ^c^				Sensitivity to Exogenic Stress ^d^			
Phenotype	Isolate	IM		WA		PDA		IM		PDA		IM		OSS		ROS	
TBZ^S^PYR^S^FDL^S^	*Pe*-40	22.4 ± 7.6	bcdef ^ef^	41.8 ± 12.2	ab	29.7 ± 2.9	bcde	33.3 ± 1.2	c	8.41 ± 0.1	ab	7.76 ± 0.1	a	9.9 ± 2.8	ab	0.0 ± 0.0	a
Wild-Type	*Pe*-1267	25.8 ± 5.6	bcde	45.6 ± 14.3	a	23.0 ± 3.3	gh	34.3 ± 1.4	abc	8.20 ± 0.2	b	7.75 ± 0.1	a	5.0 ± 3.0	de	0.0 ± 0.0	a
	*Pe*-2418	32.0 ± 7.7	abc	51.9 ± 13.0	a	33.8 ± 2.0	ab	35.8 ± 0.9	abc	8.37 ± 0.1	ab	7.74 ± 0.1	a	8.9 ± 2.3	ab	0.0 ± 0.0	a
	**Mean**	**26.7 ± 7.9**		**46.4 ± 13.6**		**28.8 ± 5.2**		**34.4 ± 5.1**		**8.33 ± 0.2**		**7.75 ± 0.1**		**8.0 ± 3.4**		**0.0 ± 0.0**	
TBZ^R^PYR^S^FDL^S^	*Pe*-23	20.0 ± 10.1	cdefg	31.3 ± 7.4	abc	23.5 ± 3.6	gh	32.8 ± 0.9	c	8.37 ± 0.1	ab	7.74 ± 0.2	a	7.5 ± 2.1	bcd	0.0 ± 0.0	a
Single-Resistant	*Pe*-184	5.6 ± 1.7	gh	14.6 ± 7.6	ef	24.8 ± 2.6	fgh	32.7 ± 1.4	c	8.36 ± 0.1	ab	7.84 ± 0.1	a	7.8 ± 1.4	bc	0.0 ± 0.0	a
	*Pe*-219	17.4 ± 12.4	defg	12.3 ± 4.0	f	29.3 ± 1.9	bcde	34.8 ± 1.0	abc	8.34 ± 0.1	ab	7.81 ± 0.1	a	5.8 ± 1.4	cde	0.0 ± 0.0	a
	**Mean**	**14.3 ± 11.0**		**19.4 ± 10.7**		**25.9 ± 3.7**		**33.4 ± 4.8**		**8.36 ± 0.1**		**7.80 ± 0.1**		**7.0 ± 1.9**		**0.0 ± 0.0**	
TBZ^R^PYR^R^FDL^S^	*Pe*-08	25.9 ± 13.6	bcde	23.8 ± 3.5	cd	23.9 ± 2.8	fgh	33.9 ± 0.6	bc	8.41 ± 0.1	ab	7.82 ± 0.1	a	10.8 ± 1.9	a	0.0 ± 0.0	a
Dual-Resistant	*Pe*-2311	6.6 ± 2.6	fgh	20.8 ± 7.7	de	26.5 ± 2.7	efgh	33.5 ± 0.5	c	8.37 ± 0.1	ab	7.89 ± 0.1	a	8.2 ± 2.3	abc	0.0 ± 0.0	a
	*Pe*-2483	1.2 ± 1.9	h	15.7 ± 6.4	ef	28.4 ± 1.5	cdef	33.8 ± 0.9	abc	8.38 ± 0.0	ab	7.77 ± 0.2	a	7.2 ± 1.8	bcd	0.0 ± 0.0	a
	**Mean**	**10.8 ± 13.2**		**20.1 ± 6.9**		**26.3 ± 3.0**		**33.8 ± 2.9**		**8.39 ± 0.1**		**7.83 ± 0.1**		**8.8 ± 2.5**		**0.0 ± 0.0**	
TBZ^S^PYR^R^FDL^R^	*Pe*-153	51.8 ± 10.1	a	20.6 ± 7.4	de	31.2 ± 6.8	abcd	35.6 ± 0.9	abc	8.44 ± 0.1	a	7.77 ± 0.1	a	4.4 ± 1.7	ef	0.0 ± 0.0	a
Dual-Resistant	*Pe*-2501	30.8 ± 9.1	abcd	29.0 ± 10.9	bcd	27.0 ± 5.8	defg	35.2 ± 2.9	abc	8.42 ± 0.1	a	7.74 ± 0.2	a	3.3 ± 3.4	efg	0.0 ± 0.0	a
	*Pe*-2517	35.7 ± 7.3	ab	10.7 ± 3.7	f	31.1 ± 5.0	abcd	33.9 ± 0.8	bc	8.46 ± 0.0	a	7.60 ± 0.2	a	9.8 ± 3.0	ab	0.0 ± 0.0	a
	**Mean**	**39.4 ± 12.5**		**20.1 ± 10.8**		**29.8 ± 6.1**		**34.9 ± 3.5**		**8.44 ± 0.1**		**7.70 ± 0.2**		**5.8 ± 4.0**		**0.0 ± 0.0**	
TBZ^R^PYR^R^FDL^R^	*Pe*-1020	31.2 ± 9.7	abcd	21.6 ± 10.3	de	22.0 ± 5.8	h	35.3 ± 1.0	abc	8.36 ± 0.1	ab	7.83 ± 0.1	a	2.1 ± 1.2	fg	0.0 ± 0.0	a
Triple-Resistant	*Pe*-2754	26.0 ± 5.4	bcde	21.6 ± 8.9	de	31.6 ± 4.2	abc	39.0 ± 1.2	a	8.44 ± 0.0	a	7.75 ± 0.1	a	1.6 ± 0.8	g	0.0 ± 0.0	a
	*Pe*-3045	14.3 ± 2.4	efgh	21.4 ± 6.4	de	34.5 ± 4.3	a	37.5 ± 0.8	ab	8.45 ± 0.1	a	7.77 ± 0.2	a	1.8 ± 1.2	fg	0.0 ± 0.0	a
	**Mean**	**23.9 ± 9.6**		**21.5 ± 8.4**		**29.4 ± 7.2**		**37.3 ± 4.4**		**8.41 ± 0.1**		**7.78 ± 0.1**		**1.8 ± 1.1**		**0.0 ± 0.0**	

^a^ Spore germination (%) was assessed after 96 h on intermediate medium (IM) and 10 days on water agar (WA). ^b^ Mycelial growth (mm) was assessed on PDA and IM after 120 days. ^c^ Sporulation (conidia mL^−1^) was measured after 120 days on PDA and IM. ^d^ Sensitivity to exogenic stress (colony diameter in mm) was assessed after 120 days on PDA amended with 12% NaCl or with 30 mM of methyl viologen for osmotic stress (OSS) and reactive oxygen species (ROS), respectively. ^e^ Mean and standard deviation are shown for each isolate, medium, and fitness parameter combination. ^f^ Differences in letters indicate statistically significant difference based on ANOVA and Tukey-HSD (all parameters except germination on IM and sporulation) or Kruskal–Wallis and Dunn’s (germination and sporulation) tests at *p* < 0.05.

**Table 5:** Cumulative fitness change (CF_C_ in %) in resistant isolates of *Penicillium expansum* with different sensitivity phenotypes relative to sensitive wild-type isolates in vitro and on apples.

				Cumulative Relative Fitness Change (CF_C_) in the Resistant Isolates to Sensitive Isolates											
		Resistance		WA ^b^		IM		PDA		On Detached Apples					
Resistance Group	Isolate	Phenotype ^a^		1.5 °C	20 °C	1.5 °C	20 °C	1.5 °C	20 °C	Control	TBZ	PYR	FDL	Fitness Gain	Fitness Loss
**Single-Resistant**	*Pe*-23	TBZ^R^		−39	−28	−34	4	−13	42	4	393	0	−200	0	0
	*Pe*-184	TBZ^R^		−104	−20	−135	0	9	70	11	393	200	267	5	−5
	*Pe*-219	TBZ^R^		−116	−13	−40	−1	−94	80	-	-	-	-	25	−25
														50	−50
			**Mean**	**−86**	**−20**	**−70**	**1**	**−33**	**64**	**7**	**393**	**100**	**33**	100	−100
														200	−200
**Dual-Resistant**	*Pe*-08	TBZ^R^PYR^R^		−64	−6	−4	4	−44	34	−3	393	400	−200		
	*Pe*-2311	TBZ^R^PYR^R^		−76	−32	−122	4	−65	38	−7	393	400	−200		
	*Pe*-2483	TBZ^R^PYR^R^		−99	−23	−184	4	−89	−6	-	-	-	-		
			**Mean**	**−80**	**−20**	**−103**	**4**	**−66**	**22**	**−5**	**393**	**400**	**−200**		
	*Pe*-153	PYR^R^FDL^R^		−77	−11	67	−1	36	−42	−23	60	0	391		
	*Pe*-2501	PYR^R^FDL^R^		−46	−11	16	0	−67	−86	−4	55	200	0		
	*Pe*-2517	PYR^R^FDL^R^		−125	−23	25	−2	17	−92	-	-	-	-		
			**Mean**	**−83**	**−15**	**36**	**−1**	**−5**	**−73**	**−14**	**57**	**100**	**196**		
**Triple-Resistant**	*Pe*-1020	TBZ^R^PYR^R^FDL^R^		−73	−33	19	7	−161	30	−5	393	400	379		
	*Pe*-2754	TBZ^R^PYR^R^FDL^R^		−73	−23	10	3	−118	−85	−10	393	400	394		
	*Pe*-3045	TBZ^R^PYR^R^FDL^R^		−71	−21	−51	2	−135	−160	-	-	-	-		
			**Mean**	**−72**	**−26**	**−8**	**4**	**−138**	**−71**	**−7**	**393**	**400**	**386**		

^a^ TBZ, PYR, and FDL indicate thiabendazole, pyrimethanil, and fludioxonil, respectively. S and R depict sensitive and resistant isolates, respectively, to specific fungicide. ^b^ WA, IM, and PDA denote water agar, intermediate medium, and potato dextrose agar, respectively. - indicates isolate not tested on detached apples. Yellow, green, and red colors indicates non-significant change, significant gain, and significant loss, respectively.

## Data Availability

The original contributions presented in this study are included in the article and Supplementary Materials. Further inquiries can be directed to the corresponding author.

## Supplementary Materials

The following supporting information can be downloaded at: https://www.mdpi.com/article/10.3390/microorganisms13081846/s1. Figure S1: Expression of *Ac1* (A), *Blistering1* (B), *OS2* (C), *RacA* (D), *BrlA* (E), *LaeA* (F), and *VeA* (G) genes relative to reference genes *28s* and *CMD* in *Penicillium expansum* of different sensitivity phenotypes after 7 days at 20 °C on PDA (control) and on PDA amended with thiabendazole (TBZ), pyrimethanil (PYR), and fludioxonil (FDL) at 0.13, 0.01, and 0.01 µg/mL, respectively. Table S1: Thermocycler conditions and primer efficiencies for fitness gene expression analysis via qPCR. Table S2: Germination and mycelial growth of 15 Penicillium isolates on different media and at various incubation periods. Table S3: Effective concentrations inhibiting 50% germination or growth (EC_50_) of thiabendazole, pyrimethanil, and fludioxonil in 15 *Penicillium expansum* isolates.

## Abbreviations

The following abbreviations are used in this manuscript:

TBZ	Thiabendazole
PYR	Pyrimethanil
FDL	Fludioxonil
TBZ^S^PYR^S^FDL^S^	Wild-type isolates sensitive to all three fungicides
TBZ^R^	Single-resistant isolate to thiabendazole
TBZ^R^PY^R^	Dual-resistant isolate to thiabendazole and pyrimethanil
PYR^R^FDL^R^	Dual-resistant isolate to pyrimethanil and fludioxonil
TBZ^R^PYR^R^FDL^R^	Triple-resistant isolates to all three fungicides
Fc	Fitness change relative to wild-type isolates
CFc	Cumulative fitness change
FRAC	Fungicide Resistance Action Committee
Gw or Gm	Generation at a given week or month following transfer of isolates
OSS	Osmotic stress
ROS	Reactive oxygen species

## References

[1] Amiri A., Bompeix G. (2005). Diversity and population dynamics of *Penicillium* spp. on apples in pre- and postharvest environments: Consequences for decay development. Plant Pathol..

[2] Rosenberger D.A., Jones A.L., Aldwinkle H.S. (1990). Blue mold. Compendium of Apple and Pear Diseases.

[3] Errampalli D., Crnko N. (2004). Control of blue mold caused by *Penicillium expansum* on apples “Empire” with fludioxonil and cyprodinil. Can. J. Plant Pathol..

[4] Wang K., Ngea G.L.N., Godana E.A., Shi Y., Lanhuang B., Zhang X., Zhao L., Yang Q., Wang S., Zhang H. (2023). Recent advances in *Penicillium expansum* infection mechanisms and current methods in controlling *P. expansum* in postharvest apples. Crit. Rev. Food Sci. Nutr..

[5] Amiri A., Ali E.M. Prevalence of Storage Decays of Apple: Lessons from the 2016 Statewide Survey. https://treefruit.wsu.edu/article/prevalence-of-storage-decays-of-apple-lessons-from-the-2016-statewide-survey/.

[6] Sanderson P.G., Spotts R.A. (1995). Postharvest decay of winter pear and apple fruit caused by species of *Penicillium*. Phytopathology.

[7] Dutoit M., Nelson L.M., Tyson R. (2013). Predicting the spread of postharvest disease in stored fruit, with application to apples. Postharvest Biol. Technol..

[8] Fungicide Resistance Action Committee Pathogen Risk List 2019. https://www.frac.info/publications/all-downloads/#open-tour.

[9] Pandey M., Amiri A. (2024). High resistance levels to pyrimethanil and fludioxonil among fourteen *Penicillium* spp. from pome fruits in the U.S. Pacific Northwest. Pestic. Biochem. Physiol..

[10] Pandey M., Haskell C.L., Cowell J.D., Amiri A. (2025). Sensitivity to the Demethylation Inhibitor Difenoconazole Among Baseline Populations of Various *Penicillium* spp. Causing Blue Mold of Apples and Pears. J. Fungi.

[11] Xiao C.L., Kim K.K., Boal R.J. (2011). First report of occurrence of pyrimethanil resistance in *Penicillium expansum* from stored apples in Washington State. Plant Dis..

[12] Baraldi E., Mari M., Chierici E., Pondrelli M., Bertolini P., Pratella G.C. (2003). Studies on thiabendazole resistance of *Penicillium expansum* of pears: Pathogenic fitness and genetic characterization. Plant Pathol..

[13] Malandrakis A.A., Markoglou A.N., Konstantinou S., Doukas E.C., Kalmpokis J.F., Karaoglanidis G.S. (2013). Molecular characterization, fitness and mycotoxin production of benzimidazole-resistant isolates of *Penicillium expansum*. Int. J. Food Microbiol..

[14] Yan H.J., Gaskins V.L., Vico I., Luo Y.G., Jurick W.M. (2014). First report of *Penicillium expansum* isolates resistant to pyrimethanil from stored apple fruit in Pennsylvania. Plant Dis..

[15] Bertrand P.F., Saulie-Carter J.L. (1978). The occurrence of benomyl-tolerant strains of *Penicillium expansum* and *Botrytis cinerea* in the mid-Columbia region of Oregon and Washington. Plant Dis. Rep..

[16] Cabañas R., Castellá G., Lourdes Abaarca M., Rosa Bragulat M., Javier Cabañes F. (2009). Thiabendazole resistance and mutations in the ß-tubulin gene of *Penicillium expansum* strains isolated from apples and pears with blue mold decay. FEMS Microbiol. Lett..

[17] Yin Y.N., Xiao C.L. (2013). Molecular characterization and a multiplex allele-specific PCR method for detection of thiabendazole resistance in *Penicillium expansum* from apple. Eur. J. Plant Pathol..

[18] Kanetis L., Forster H., Jones C.A., Borkovich K.A., Adaskaveg J.E. (2008). Characterization of genetic and biochemical mechanisms of fludioxonil and pyrimethanil resistance in field isolates of *Penicillium digitatum*. Phytopathology.

[19] Zhang Y., Fu Y., Luo C., Zhu F. (2021). Pyrimethanil sensitivity and resistance mechanisms in *Penicillium digitatum*. Plant Dis..

[20] Fritz R., Lanen C., Colas V., Leroux P. (1997). Inhibition of methionine biosynthesis in *Botrytis cinerea* by the anilinopyrimidine fungicide pyrimethanil. Pestic. Sci..

[21] Mosbach A., Edel D., Farmer A.D., Widdison S., Barchietto T., Dietrich R.A., Corran A., Scalliet G. (2017). Anilinopyrimidine resistance in *Botrytis cinerea* is linked to mitochondrial function. Front. Microbiol..

[22] Amiri A., Ali M.E., De Angelis D.R., Mulvaney K.A., Pandit L.K. (2021). Prevalence and distribution of *Penicillium expansum* and *Botrytis cinerea* in apple packinghouses across Washington State and their sensitivity to the postharvest fungicide pyrimethanil. Acta Hortic..

[23] Karaoglanidis G.S., Markoglou A.N., Bardas G.A., Doukas E.G., Konstantinou S., Kalampokis J.F. (2011). Sensitivity of *Penicillium expansum* field isolates to tebuconazole, iprodione, fludioxonil and cyprodinil and characterization of fitness parameters and patulin production. Int. J. Food Microbiol..

[24] Samaras A., Ntasiou P., Myresiotis C., Karaoglandis G. (2020). Multidrug resistance of *Penicillium expansum* to fungicides: Whole transcriptome analysis of MDR strains reveals overexpression of efflux transporter genes. Int. J. Food Microbiol..

[25] Gandia M., Garrigues S., Hernanz-Koers M., Manzanares P. (2019). Differential roles, crosstalk and response to the antifungal protein AfpB in the three mitogen-activated protein kinases (MAPK) pathways of the citrus postharvest pathogen *Penicillium digitatum*. Fungal Genet. Biol..

[26] Zhang Y., Lamm R., Pillonel C., Lam S., Xu J.-R. (2002). Osmoregulation and fungicide resistance: The *Neurospore crassa os-2* gene encodes a *HOG1* mitogen-activated protein kinase homologue. Appl. Environ. Microbiol..

[27] Gaskins V.L., Yu I.V., Jurick W.M. (2015). First Report of *Penicillium expansum* isolates with reduced sensitivity to fludioxonil from a commercial packinghouse in Pennsylvania. Plant Dis..

[28] Oiki S., Yaguchi T., Urayama S.I., Hagiwara D. (2022). Wide distribution of resistance to the fungicides fludioxonil and iprodione in *Penicillium* species. PLoS ONE.

[29] Wang M., Chen C., Zhu C., Sun X., Ruan R. (2014). *Os2* MAP kinase-mediated osmostress tolerance in *Penicillium digitatum* is associated with its positive regulation on glycerol synthesis and negative regulation on ergosterol synthesis. Microbiol. Res..

[30] Gilchrist M.A., Sulsky D.L., Pringle A. (2006). Identifying fitness and optimal life-history strategies for an asexual filamentous fungus. Evolution.

[31] Li H.X., Xiao C.L. (2008). Characterization of fludioxonil-resistant and pyrimethanil-resistant phenotypes of *Penicillium expansum* from apple. Phytopathology.

[32] El Hajj Assaf C., Snini S.P., Tadrist S., Bailly S., Naylies C., Oswald I.P., Lorber S., Puel O. (2018). Impact of veA on the development, aggressiveness, dissemination and secondary metabolism of *Penicillium expansum*. Mol. Plant Pathol..

[33] Kumar D., Barad S., Chen Y., Luo X., Tannous J., Dubey A., Matana N., Tian S., Li B., Keller N. (2017). LaeA regulation of secondary metabolism modulates virulence in *Penicillium expansum* and is mediated by sucrose. Mol. Plant Pathol..

[34] Jurick W.M., Peng H., Beard H.S., Garrett W.M., Lichtner F.J., Luciano-Rosario D., Macarisin O., Liu Y., Peter K.A., Gaskins V.L. (2020). Blistering1 modulates *Penicillium expansum* virulence via vesicle-mediated protein secretion. Mol. Cell. Proteom..

[35] Zetina-Serrano C., Rocher O., Naylies C., Lippi Y., Oswald I.P., Lorber S., Purl O. (2020). The brlA gene deletion reveals that patulin biosynthesis is not related to conidiation in *Penicillium expansum*. Int. J. Mol. Sci..

[36] Wang W., Wang M., Wang J., Zhu C., Chung K.-R., Li H. (2016). Adenylyl cyclase is required for cAMP production, growth, conidial germination, and virulence in the citrus green mold pathogen *Penicillium digitatum*. Microbiol. Res..

[37] Rodríguez A., Medina Á., Córdoba J.J., Magan N. (2016). Development of a HOG-based real-time PCR method to detect stress response changes in mycotoxigenic moulds. Food Microbiol..

[38] Monteau S., Abouna S., Lambert B., Legendre L. (2003). Differential regulation by ambient pH of putative virulence factor secretion by the phytopathogenic fungus *Botrytis cinerea*. FEMS Microbiol. Ecol..

[39] Zhang X., Zong Y., Gong D., Yu L., Sionov E., Bi Y., Prusky D. (2021). NADPH oxidase regulates the growth and pathogenicity of *Penicillium expansum*. Front. Plant Sci..

[40] Visagie C.M., Houbraken J., Frisvad J.C., Hong S.-B., Klaassen C.H.W., Perrone G., Seifert K.A., Varga J., Yaguchi T., Samson R.A. (2014). Identification and nomenclature of the genus *Penicillium*. Stud. Mycol..

[41] Cabañas R., Abarca M.L., Bragulat M.R., Cabañes F.J. (2009). Comparison of methods to detect resistance of *Penicillium expansum* to thiabendazole. Lett. Appl. Microbiol..

[42] Li H.X., Xiao C.L. (2008). Baseline sensitivities to fludioxonil and pyrimethanil in *Penicillium expansum* populations from apple in Washington State. Postharvest Biol. Technol..

[43] Ali E.M., Amiri A. (2018). Selection pressure pathways and mechanisms of resistance to demethylation inhibitor-Difenoconazole in *Penicillium expansum*. Front. Microbiol..

[44] Vandesompele J., De Preter K., Pattyn F., Poppe B., Van Roy N., De Paepe A., Speleman F. (2002). Accurate normalization of real-time quantitative RT-PCR data by geometric averaging of multiple internal control genes. Genome Biol..

[45] Hellemans J., Mortier G., De Paepe A., Speleman F., Vandesompele J. (2007). qBase relative quantification framework and software for management and automated analysis of real-time quantitative PCR data. Genome Biol..

[46] Cole T.J., Altman D.G. (2017). Statistics notes: What is a percentage difference?. BMJ.

[47] Markoglou A.N., Doukas E.G., Malandrakis A.A. (2011). Effect of anilinopyrimidine resistance on aflatoxin production and fitness parameters in *Aspergillus parasiticus* Speare. Int. J. Food Microbiol..

[48] Hiber U.W., Schüepp H., Schwinn F.J., Heaney S., Slawson D., Hollomon D.W., Smith M., Russel P.E., Perry D.W. (1994). Resistance risk evaluation of fludioxonil, a new phenylpyrrole fungicide. Fungicide Resistance, Monograph No.60.

[49] Tudzynski P., Heller J., Siegmund U. (2012). Reactive oxygen species generation in fungal development and pathogenesis. Curr. Opin. Microbiol..

[50] Bilsland E., Molin C., Swaminathan S., Ramne A., Sunnerhagen P. (2004). Rck1 and Rck2 MAPKAP kinases and the HOG pathway are required for oxidative stress resistance. Mol. Microbiol..

[51] Segmüller N., Ellendorf U., Tudzynski B., Tudzynski P. (2007). BcSAK1, a stress-activated mitogen-activated protein kinase, is involved in vegetative differentiation and pathogenicity in *Botrytis cinerea*. Eukaryot. Cells.

[52] Zhang T., Cao Q., Li N., Liu D., Yuan Y. (2020). Transcriptome analysis of fungicide-responsive gene expression profiles in two *Penicillium italicum* strains with different responses to the sterol demethylation inhibitor (DMI) fungicide prochloraz. BMC Genom..

[53] Hawkins N.J., Fraaije B.A. (2018). Fitness penalties in the evolution of fungicide resistance. Annu. Rev. Phytopathol..

